# The presentation, diagnosis and management of non-traumatic wrist pain: an evaluation of current practice in secondary care in the UK NHS

**DOI:** 10.1093/rap/rkaa030

**Published:** 2020-07-07

**Authors:** The MOWP study group:, Benjamin J F Dean, Andrew Carr, Ryan W Trickett, Stefan Kluzek, Nicholas Riley, Christopher P Bretherton, Melanie K Wilson, Mike J Hayton, Neal R Rupani, Ching Cheng Daniel Hsieh, Laura J Clifton, Peter Dacombe, Lydia K Milnes, Raveen L Jayasuriya, Harvey A George, Rishi Das, Alistair Mayne, Matthew T Brown, Stephen J Lipscombe, Gillian L Eastwood, Richard M Unsworth, Lucie J Wright, Mohammed As-Sultany

**Affiliations:** r1 Nuffield Department of Orthopaedics, Rheumatology and Musculoskeletal Sciences (NDORMS), Botnar Research Centre, Oxford; r2 Department of Orthopaedic Surgery, Nuffield Orthopaedic Centre, Oxford University Hospitals NHS Foundation Trust, Oxford; r3 Department of Orthopaedic Surgery, Cardiff & Vale University Health Board, Cardiff; r4 Department of Orthopaedic Surgery, Nottingham University Hospitals, Nottingham; r5 Department of Orthopaedic Surgery, Nottingham University, The Queen’s Medical Centre, Nottingham; r6 Department of Orthopaedic Surgery, Northwick Park Hospital, Harrow; r7 Department of Orthopaedic Surgery, Wrightington, Wigan and Leigh NHS Trust, Wigan; r8 Department of Orthopaedic Surgery, Frimley Health NHS Foundation Trust, Camberley; r9 Department of Orthopaedic Surgery, University of Buckingham Medical School, Buckingham; r10 Department of Orthopaedic Surgery, Royal Berkshire Hospital, Reading; r11 Department of Orthopaedic Surgery, Great Western Hospital, Swindon; r12 Department of Orthopaedic Surgery, Chelsea and Westminster Hospital, London; r13 Department of Orthopaedic Surgery, Sheffield Teaching Hospitals NHS Foundation Trust, Royal Hallamshire Hospital, Sheffield; r14 Department of Orthopaedic Surgery, North Hampshire Hospitals NHS FT, Basingstoke; r15 Department of Orthopaedic Surgery, Craigavon Area Hospital, Craigavon; r16 Department of Orthopaedic Surgery, The Princess Alexandra Hospital NHS Trust, Harlow; r17 Department of Orthopaedic Surgery, Whiston Hospital, Prescot; r18 Department of Orthopaedic Surgery, Stockport NHS Foundation Trust, Stockport, UK

**Keywords:** wrist, pain, osteoarthritis, surgery, National Health Service

## Abstract

**Objectives:**

The study aims were to assess the burden of non-traumatic wrist pain in terms of numbers of referrals to secondary care and to characterize how patients present, are diagnosed and are managed in secondary care in the UK National Health Service.

**Methods:**

Ten consecutive patients presenting with non-traumatic wrist pain were identified retrospectively at each of 16 participating hospitals, and data were extracted for 12 months after the initial referral.

**Results:**

The 160 patients consisted of 100 females and 60 males with a median age of 49 years, accounting for ∼13% of all new hand/wrist referrals. The dominant wrist was affected in 60% of cases, and the mean symptom duration was 13.3 months. Diagnoses were grouped as follows: OA (31%), tendinopathy (13%), ganglion (14%), ulnar sided pain (17%) and other (25%). The OA group was significantly older than other groups, and other groups contained a predominance of females. The non-surgical interventions, in decreasing frequency of usage, were as follows: CS injections (39%), physiotherapy (32%), splint (31%) and analgesics (12%). Of those who underwent surgery, all patients had previously received non-surgical treatment, but 42% had undergone only one non-surgical intervention.

**Conclusions:**

Non-traumatic wrist pain represents a significant burden to secondary care both in terms of new patient referrals and in terms of investigation, follow-up and treatment. Those presenting with OA are more likely to be older and male, whereas those presenting with other diagnoses are more likely to be younger and female.

**Key messages**

- Non-traumatic wrist pain represents a significant burden to secondary care in the UK.
- The most common diagnostic group was OA of the wrist.
- The most widely used non-surgical intervention was the CS injection.

## Introduction

Wrist pain is a common problem, accounting for an annual consultation prevalence rate of 58 in 10 000 patients in primary care in the UK [1]; ∼1/10th of the consultation rate for back pain, the most common site of musculoskeletal pain. The prevalence of non-specific hand and wrist pain is ∼10% in the general population [2], higher than the combined total prevalence of de Quervain’s tenosynovitis, wrist tenosynovitis and carpal tunnel syndrome reported at ∼3%. Wrist pain is more prevalent in those who work in more physically demanding occupations and in sportspeople [3].

The variable structure of local health-care systems within the National Health Service (NHS) in the UK means that pathways for non-traumatic wrist pain are likely to be heterogeneous [4]. Generally, referrals pass from primary care, through an interface musculoskeletal service for initial diagnostics and treatment, with secondary care referrals emerging as necessary. Relatively little has been published regarding the presentation, diagnosis and management of non-traumatic wrist pain in both interface and secondary care services.

In this context, the specific aims of this study were to assess the overall proportion of referrals for non-traumatic wrist pain received by specialist hand and wrist clinics in the UK, to describe the demographics and diagnoses in these patients and to describe the investigations and interventions performed.

## Methods

Ten consecutive patients presenting with non-traumatic wrist pain were identified from specialist hand and wrist clinics in 16 UK hospitals. Data collection was performed collaboratively, using orthopaedic higher surgical trainees and consultants (invited via the British Orthopaedic Network Environment) and informal, regional consultant networks. No hospitals were excluded. Data gathering was approved via the audit department of each participating hospital.

**Fig. 1:**
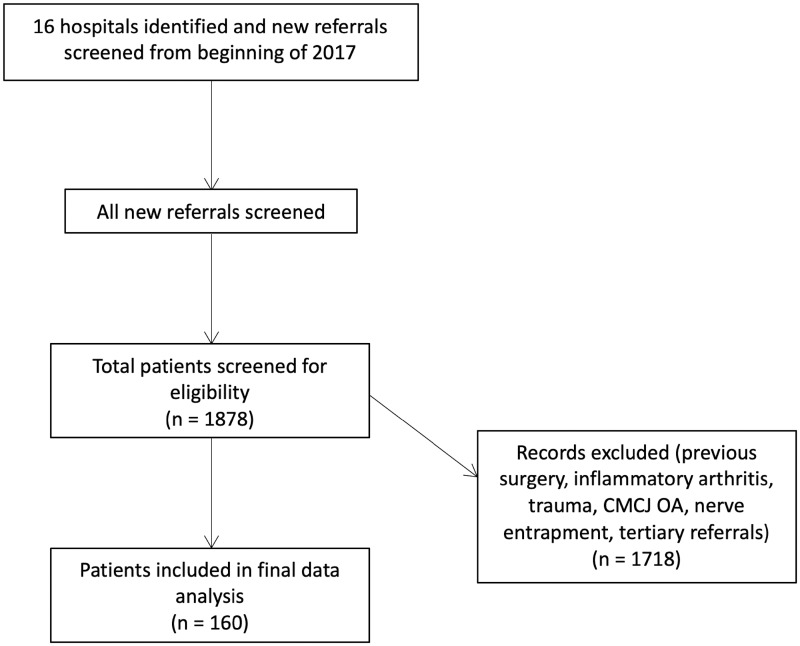
Flow diagram demonstrating patient selection process

Patients were identified retrospectively by reviewing all new patient referrals from 1 January 2017 onwards. Patients with a clear history or radiological evidence of substantial trauma were excluded (i.e. scaphoid fracture or non-union/distal radius fracture or malunion/fracture clinic patients), as were patients with previous wrist surgery to the affected side, diagnosis of inflammatory arthritis, a suspected diagnosis of carpal tunnel syndrome, thumb base degeneration or patients referred by another hand/wrist specialist for a second/third opinion. Fig. 1 summarizes the patient selection process.

The first 10 patients presenting with non-traumatic wrist pain were reviewed in detail from first appointment through to discharge or to 12 months after the initial appointment, whichever occurred first. The total number of new patient referrals required to obtain 10 non-traumatic wrist pains was recorded. Data were collected using a standardized form, including age, biological sex, hand dominance, employment status, date of first appointment, site of wrist pain, duration of symptoms, investigations undertaken, non-surgical interventions undertaken, final stated diagnosis, date and type of surgery, complications, the number of appointments over the 1 year period and whether the patient had been discharged by the end of this year. If diagnostic uncertainty remained at the end of follow-up, this was recorded as unknown. The data related to the clinical documentation only. Thus, if use of analgesia had not been documented specifically, for the purposes of this study it did not occur.

Five broad diagnostic categories were generated by consensus involving the senior surgeons within the group (N.R. and R.W.T.) before data analysis: OA, including radiocarpal, midcarpal or distal radioulnar joint; tendinopathy; ulnar sided pain, including ulnocarpal abutment, triangular fibrocartilage and extensor carpi ulnaris pathology; ganglion; and other (non-traumatic instability, avascular necrosis, non-specific and unknown).

### Statistics

Statistical analysis was carried out using GraphPad Prism v.5.00 for Windows (GraphPad Software, San Diego, CA, USA; www.graphpad.com) and with STATA/IC v.16 (StataCorp LP, College Station, TX, USA). Histograms for all data sets were analysed to assess for normality. Data were normally distributed unless stated otherwise. Results are expressed as the mean (s.d.) unless stated otherwise. Student’s unpaired *t*-tests and Mann–Whitney *U*-tests were used to test for differences between two groups for parametric and non-parametric data, respectively. The Kruskal–Wallis one-way ANOVA was used to test for differences within multiple groups of non-parametric data, and ANOVA was used to test multiple groups of parametric data. Fisher’s exact test was used to test for differences between two categorical variables. Statistical significance was set at a level of *P* < 0.05.

## Results

### Centres, referral patterns and burden

The details relating to the 16 participating centres are presented in Supplementary Table S1. The mean proportion of hand and wrist clinic referrals that related to non-traumatic wrist pain was 12.9% over a mean 106-day review period. Using this proportion, correcting for the observed 106-day review period and assuming a UK population of 66 million, there are ∼4228 new patient referrals to secondary care for non-traumatic wrist pain per annum in the UK (Fig. 1).

### Patient demographics and characteristics

There were 100 females and 60 males, with a median age of 49 (interquartile range 34–60) years. The dominant wrist was affected in 60% of cases, and the mean symptom duration was 13.3 months. OA of the radiocarpal or midcarpal joints was the most common diagnosis within the nine categories considered (Table 1). A further breakdown of the diagnoses within each diagnostic group is detailed in Supplementary Table S2.

**Table 1:** Breakdown of patient demographics and characteristics based on gender

Factor	Level	All	Male	Female	*P*-value (between male and female)
*n*		160	60	100	
Age, median (interquartile range), years		49.0 (34.0–60.0)	54.5 (34.0–68.5)	47.5 (33.0–54.0)	0.035*
Wrist affected, *n* (%)	Dominant	96 (60.0)	33 (55.0)	63 (63.0)	0.47
	Non-dominant	52 (32.5)	23 (38.3)	29 (29.0)	
	Both	12 (7.5)	4 (6.7)	8 (8.0)	
Diagnosis, *n* (%)	OA	49 (30.6)	34 (56.7)	15 (15)	<0.001***
	Ulnar group	28 (17.5)	7 (11.7)	21 (21.0)	
	Tendinopathy	21 (13.1)	4 (6.7)	17 (17.0)	
	Ganglion	22 (13.8)	2 (3.3)	20 (20.0)	
	Other	40 (25)	13 (21.7)	27 (27)	
Symptom duration, mean (s.d.), months		13.3 (11.3)	14.4 (12.4)	12.6 (10.6)	0.33
Site of wrist pain, *n* (%)	Ulnar	46 (28.7)	19 (31.7)	27 (27.0)	0.50
	Radial	47 (29.4)	18 (30.0)	29 (29.0)	
	Central	39 (24.4)	16 (26.7)	23 (23.0)	
	Diffuse	28 (17.5)	7 (11.7)	21 (21.0)	
Employment, *n* (%)	Unemployed	12 (7.5)	1 (1.7)	11 (11.0)	0.032*
	Employed	92 (57.5)	39 (65.0)	53 (53.0)	
	Retired	27 (16.9)	13 (21.7)	14 (14.0)	
	Unknown	29 (18.1)	7 (11.7)	22 (22.0)	

***P-value < 0.001, **P-value < 0.01, *P-value < 0.05.

Patients with a diagnosis of OA were significantly older (median age 64 years) than other groups (median ages between 41 and 44 years) and contained a statistically significant predominance of males (69%, *P* < 0.001; Table 2). There was a similar proportion of dominant (49%) and non-dominant (45%) wrists affected. In comparison, the other diagnostic groups were predominantly female (between 67 and 91%), with the dominant wrist affected (between 57 and 73%; *P* = 0.09).

**Table 2:** Breakdown of patient demographics and characteristics based on the five diagnostic groups

Factor	Level	OA	Tendinopathy	Ganglion	Ulnar group	Other	*P*-value
Number of patients, *n* (%)		49 (31)	21 (13)	22 (14)	28 (17)	40 (25)	
Age, median (interquartile range), years		64.0 (53.0–73.0)	44.0 (37.0–54.0)	41.0 (27.0–53.0)	44.0 (32.0–51.0)	44.0 (30.0–52.0)	<0.001***
Sex, *n* (%)	Male	34 (69)	4 (19)	2 (9)	7 (25)	13 (33)	<0.001***
	Female	15 (31)	17 (81)	20 (91)	21 (75)	27 (68)	
Wrist affected, *n* (%)	Dominant	24 (49)	13 (62)	16 (73)	16 (57)	27 (68)	0.089
	Non-dominant	22 (45)	4 (19)	6 (27)	11 (39)	9 (23)	
	Both	3 (6)	4 (19)	0 (0)	1 (4)	4 (10)	
Symptom duration, mean (s.d.), months		13.8 (10.9)	11.5 (10.3)	11.4 (7.1)	15.6 (12.5)	13.1 (13.3)	0.66
Site, *n* (%)	Ulnar	12 (24)	0 (0)	2 (9)	23 (82)	9 (23)	<0.001***
	Radial	18 (37)	18 (86)	3 (14)	1 (4)	7 (18)	
	Central	10 (20)	3 (14)	10 (45)	1 (4)	15 (38)	
	Diffuse	9 (18)	0 (0)	7 (32)	3 (11)	9 (23)	
Employed, *n* (%)	Unemployed	5 (10)	1 (5)	2 (9)	1 (4)	3 (8)	0.012*
	Employed	21 (43)	11 (52)	11 (50)	21 (75)	28 (70)	
	Retired	17 (35)	2 (10)	3 (14)	3 (11)	2 (5)	
	Unknown	6 (12)	7 (33)	6 (27)	3 (11)	7 (18)	
Instability, * n* (%)	No	45 (92)	21 (100)	22 (100)	27 (96)	33 (83)	0.11
	Yes	4 (8)	0 (0)	0 (0)	1 (4)	7 (18)	

***P-value < 0.001, **P-value < 0.01, *P-value < 0.05.

### Investigations and clinical follow-up

There were statistically significant differences between diagnostic groups and investigations obtained (Table 3). Plain radiographs were the most commonly obtained investigation overall (89%) and used most frequently where an eventual diagnosis of OA was made (98%). MRI was frequently used for patients with ulnar sided (82%) and other pathology (60%) groups. US was used in 21% of patients overall and was used most frequently in tendinopathy (57%) and ganglia (32%). Use of MRI but not US was associated with a significant likelihood of being discharged within the year (*P* = 0.003). Not having an X-ray was positively associated with being discharged (*P* = 0.0001). Neither of those diagnostic modalities was associated with a decreased need for surgery (*P* = 0.3). Nerve conduction studies were used infrequently (8% overall). The median number of appointments within the 12-month period was two, with most patients having two or more appointments within the year (84%). Having more than two appointments was significantly associated with risk of having surgery (*P* = 0.0001). The proportion discharged by the end of the 12-month period was significantly different between diagnostic groups, with the group most likely to be discharged being tendinopathy (76%) and the least likely patients with ulnar sided pain (32%).

**Table 3:** Details of investigations and non-surgical interventions undertaken

Factor	Level	Overall	OA	Tendinopathy	Ganglion	Ulnar group	Other	*P*-value
Number of patients		160	49	21	22	28	40	
Investigations, *n* (%)								
X-ray	No	17 (11)	1 (2)	8 (38)	6 (27)	2 (7)	3 (8)	<0.001***
	Yes	143 (89)	48 (98)	13 (62)	16 (73)	26 (93)	37 (93)	
US	No	126 (79)	47 (96)	9 (43)	15 (68)	24 (86)	31 (79)	<0.001***
	Yes	34 (21)	2 (4)	12 (57)	7 (32)	4 (14)	9 (22)	
Nerve conduction study	No	147 (92)	49 (100)	21 (100)	19 (86)	27 (96)	31 (78)	<0.001***
	Yes	13 (8)	0 (0)	0 (0)	3 (14)	1 (4)	9 (23)	
MRI	No	89 (56)	33 (67)	19 (90)	16 (73)	5 (18)	16 (40)	<0.001***
	Yes	71 (44)	16 (33)	2 (10)	6 (27)	23 (82)	24 (60)	
Non-surgical, *n* (%)								
Analgesia	No	140 (88)	39 (80)	18 (86)	21 (95)	25 (89)	37 (93)	0.27
	Yes	20 (12)	10 (20)	3 (14)	1 (5)	3 (11)	3 (8)	
Physical therapy	No	109 (68)	32 (65)	17 (81)	21 (95)	18 (64)	21 (54)	0.01*
	Yes	51 (32)	17 (35)	4 (19)	1 (5)	10 (36)	19 (46)	
Splint	No	110 (69)	25 (51)	16 (76)	20 (91)	22 (79)	27 (68)	0.007**
	Yes	50 (31)	24 (49)	5 (24)	2 (9)	6 (21)	13 (33)	
Injection	No	97 (61)	25 (51)	9 (43)	15 (68)	17 (61)	31 (78)	0.041*
	Yes	63 (39)	24 (49)	12 (57)	7 (32)	11 (39)	9 (23)	
Any treatment	0	30 (23)	3 (6)	5 (24)	6 (27)	8 (29)	8 (21)	0.080
	1	130 (77)	46 (94)	16 (76)	16 (73)	20 (71)	32 (79)	
Number of non-surgical treatments	0	30 (19)	3 (6)	5 (24)	6 (27)	8 (29)	8 (21)	0.14
	1	52 (33)	19 (39)	9 (43)	12 (55)	7 (25)	15 (38)	
	2	46 (29)	16 (33)	3 (14)	4 (18)	9 (32)	14 (36)	
	3	14 (9)	8 (16)	3 (14)	0 (0)	2 (7)	1 (3)	
	4	7 (4)	2 (4)	1 (5)	0 (0)	2 (7)	2 (5)	
	5	1 (1)	1 (2)	0 (0)	0 (0)	0 (0)	0 (0)	
Number of appointments	0	1 (1)	1 (2)	0 (0)	0 (0)	0 (0)	0 (0)	0.40
	1	34 (21)	11 (22)	5 (24)	7 (32)	2 (7)	9 (23)	
	2	65 (41)	19 (39)	12 (57)	10 (45)	11 (39)	13 (33)	
	3	39 (24)	12 (24)	3 (14)	5 (23)	10 (36)	9 (23)	
	4	20 (13)	5 (10)	1 (5)	0 (0)	5 (18)	9 (23)	
	5	1 (1)	1 (2)	0 (0)	0 (0)	0 (0)	0 (0)	
Discharged with the first 12 months	No	81 (51)	28 (57)	5 (24)	9 (41)	19 (68)	20 (50)	0.026^*^
	Yes	79 (49)	21 (43)	16 (76)	13 (59)	9 (32)	20 (50)	

***P-value < 0.001, **P-value < 0.01, *P-value < 0.05.

### Non-surgical interventions

Non-surgical interventions included CS injections (39%), physiotherapy (32%), splint (31%) and analgesics (12%) (Table 3). Splints were used variably between diagnostic groups and most frequently in OA (49%). Physiotherapy was used differently between diagnostic groups (*P* = 0.01) and most frequently in the other group (49%). Forty-three per cent of patients were treated with two or more non-surgical interventions. Of the 51 patients receiving physiotherapy, the most common regimen was strengthening (14 patients) and unspecified (14 patients); the other regimens were as follows: activity modification (nine patients), range of motion exercises (five patients), strengthening and activity modification (three patients), splint and activity modification (two patients), splint and range of motion exercise (two patients), splint/activity modification/strengthening (one patient) and manual therapy/activity modification/range of motion exercises (one patient).

### Surgery and risk factors for surgery

Overall, 27% of patients underwent surgical intervention. In 17 of these 43 patients, a component of the primary surgery was diagnostic. The proportion of diagnostic surgery was significantly different between diagnostic groups (*P* = 0.002), with diagnostic surgery being most frequent in the ulnar sided (20%) and other (25%) groups (Table 4).

**Table 4:** Details of surgery undertaken within the different diagnostic groups

Factor	Level	Overall	OA	Tendinopathy	Ganglion	Ulnar group	Other	*P*-value
Number of patients		43	13	4	9	9	8	
Surgery, *n* (%)	No	117 (73)	36 (73)	17 (81)	13 (59)	19 (68)	32 (80)	0.38
	Yes	43 (27)	13 (27)	4 (19)	9 (41)	9 (32)	8 (20)	
Diagnostic surgery, *n* (%)	No	143 (89)	47 (96)	21 (100)	22 (100)	32 (80)	21 (75)	0.002**
	Yes	17 (11)	2 (4)	0 (0)	0 (0)	8 (20)	7 (25)	
Secondary surgery, *n* (%)	No	38 (88)	11 (85)	4 (100)	9 (100)	7 (78)	7 (88)	0.19
	Yes	5 (12)	2 (15)	0 (0)	0 (0)	2 (22)	1 (12)	
Complications, *n* (%)	No	38	13 (100)	4 (100)	9 (100)	5 (56)	7 (88)	0.056
	Yes	5 (12)	0 (0)	0 (0)	0 (0)	4 (44)	1 (12)	
Type of primary surgery, *n* (%)	Arthroscopy	9	3 (23)	0 (0)	0 (0)	4 (44)	2 (25)	<0.001***
	Arthrodesis	4	4 (31)	0 (0)	0 (0)	0 (0)	0 (0)	
	Arthroplasty including excision	4	4 (31)	0 (0)	0 (0)	0 (0)	0 (0)	
	Tendon decompression or debridement	5	0 (0)	4 (100)	0 (0)	1 (11)	0 (0)	
	Ganglion excision	9	0 (0)	0 (0)	9 (100)	0 (0)	0 (0)	
	Soft tissue reconstruction	4	0 (0)	0 (0)	0 (0)	0 (0)	4 (50)	
	Bone grafting	2	0 (0)	0 (0)	0 (0)	1 (11)	1 (13)	
	Osteotomy, ulnar	3	0 (0)	0 (0)	0 (0)	3 (33)	0 (0)	
	Other	3	2 (15)	0 (0)	0 (0)	0 (0)	1 (13)	
Type of secondary surgery, *n* (%)	Arthrodesis	3	1 (50)			1 (50)	1 (100)	0.71
	Other		1 (50)			1 (50)		

***P-value < 0.001, **P-value < 0.01, *P-value < 0.05.

Surgery was less likely in patients who had not received non-surgical treatments (*P* = 0.002; Table 5). There was no association between CS injection and subsequent surgery. There were five complications of surgery (one infection, two failed bone grafting, one instability and one broken screw), of which three required secondary surgery (one failed bone graft converted to arthrodesis, one removal of broken screw and one thumb basal joint stabilization after Brunelli procedure). The other two cases of secondary surgery consisted of arthrodesis after diagnostic arthroscopies.

**Table 5:** The relationship between surgery and investigations/interventions

Factor	Level	No	Yes	*P*-value
Surgery, *n*		117	43	
US, *n* (%)	No	92 (80.0)	33 (76.7)	0.65
	Yes	23 (20.0)	10 (23.3)	
MRI, *n* (%)	No	66 (56.9)	22 (51.2)	0.52
	Yes	50 (43.1)	21 (48.8)	
X-ray, *n* (%)	No	14 (12.0)	6 (14.0)	0.74
	Yes	103 (88.0)	37 (86.0)	
Nerve conduction study, *n* (%)	No	105 (89.7)	42 (97.7)	0.10
	Yes	12 (10.3)	1 (2.3)	
Number of investigations, mean (s.d.)		1.6 (0.8)	1.6 (0.7)	0.80
Analgesia, *n* (%)	No	103 (88.0)	37 (86.0)	0.74
	Yes	14 (12.0)	6 (14.0)	
Therapy, *n* (%)	No	78 (67.2)	31 (72.1)	0.56
	Yes	38 (32.8)	12 (27.9)	
Injection, *n* (%)	No	67 (57.3)	30 (69.8)	0.15
	Yes	50 (42.7)	13 (30.2)	
Splint, *n* (%)	No	77 (65.8)	33 (76.7)	0.19
	Yes	40 (34.2)	10 (23.3)	
Number of non-surgical treatments, *n* (%)	0	30 (25.9)	0 (0.0)	0.002**
	1	44 (37.9)	18 (41.9)	
	2	31 (26.7)	15 (34.9)	
	3	8 (6.9)	5 (11.6)	
	4	3 (2.6)	4 (9.3)	
	5	0 (0.0)	1 (2.3)	

***P-value < 0.001, **P-value < 0.01, *P-value < 0.05.

## Discussion

Like post-traumatic wrist pain [5, 6], based the findings from this sample it appears that non-traumatic wrist pain represents a significant demand on secondary care services regarding new patient referrals and the burden of investigations, follow-up and treatment. These patients generate costs to the health service in terms of investigations (44% undergo an MRI scan), non-surgical treatments, clinic time and surgery (27% undergo some form of surgical intervention).

Perhaps unsurprisingly, those patients ultimately diagnosed with OA were more likely to be older and male, whereas those having other non-osteoarthritic diagnoses were more likely to be younger and female. This is consistent with previous epidemiological research [7, 8].

Many structural abnormalities have been demonstrated to be highly prevalent in asymptomatic patients, such as those relating to the triangular fibrocartilage [9], extensor carpi ulnaris tendon [10] and ganglia [11, 12]. Given the lack of real-world data relating to commonly presenting wrist pain conditions, our study findings detailing the main diagnostic groups are of interest [OA (31%), tendinopathy (13%), ganglion (14%), ulnar including abutment/triangular fibrocartilage (18%) and other (25%)]. Given the absence of high-quality evidence relating to common wrist disorders, this points to the importance of generating high-quality evidence in order to guide practice better in this area, particularly relating to wrist OA [3]. Having an MRI scan, which was most often used for ulnar side pain, was associated with discharge within the first year.

Of those who underwent surgery, all patients had previously received non-surgical treatment, but 42% underwent only one non-surgical treatment, with the remainder being treated with two or more non-surgical therapies. This demonstrates that most patients are undergoing a reasonable course of non-surgical management before converting to a surgical option. It is notable that only 12% of patients were documented to have trialled analgesia and, although this figure is likely to be a significant underestimate of the real proportion of patients taking analgesics. In the context of the Montgomery ruling it is vital to document adequately the non-surgical interventions, such as analgesia, particularly in those undergoing surgical intervention [13].

There are limitations to this work. The methods of sampling used have resulted in potential biases; for example, the sample of hospitals might not be fully representative of the UK, and this might have had some influence on the results. The data have come from sources that rely on clear and complete documentation and are therefore exposed to potential inaccuracies. For the purpose of the analyses, we have grouped the diagnoses into broad categories, and there will be debate that this categorization could have been undertaken differently. Furthermore, given the lack of high-quality studies investigating diagnostic accuracy around the wrist, it cannot be claimed that our decision to take the final stated diagnosis as accurate is free of limitations. However, we feel that this is a pragmatic decision, serving as a sensible starting point upon which further research can be based. Given the paucity of published real-world data relating to non-traumatic wrist pain, this study provides genuinely novel information with significant clinical meaning.

### Conclusions

Non-traumatic wrist pain represents a significant burden to secondary care, both in terms of new patient referrals and in terms of investigation, follow-up and treatment. Those presenting with OA are more likely to be older and male, whereas those presenting with other diagnoses are more likely to be younger and female. Given the absence of high-quality evidence relating to common wrist disorders, this study points to the importance of generating high-quality evidence in order to guide practice in this area better, particularly in relationship to wrist OA.

## Acknowledgements

All authors have made substantial contributions to all three of sections (1), (2) and (3): (1) the conception and design of the study, or acquisition of data, or analysis and interpretation of data; (2) drafting the article or revising it critically for important intellectual content; and (3) final approval of the version to be submitted.

*Funding*: No funding has been received for this work.

*Disclosure statement*: The authors have declared no conflicts of interest.

## Supplementary data

Supplementary data are available at *Rheumatology* online.

## Supplementary Material

rkaa030_Supplementary_Data
