# Supplementary material for: The presentation, diagnosis and management of non-traumatic wrist pain: an evaluation of current practice in secondary care in the UK NHS
Source: Rheumatol Adv Pract. 2020 Jul 7;4(2):rkaa030. doi: 10.1093/rap/rkaa030 (PMC7566494; doi:10.1093/rap/rkaa030)
Supplement: rkaa030_Supplementary_Data [file rkaa030_supplementary_data.docx]

**SUPPLEMENTARY MATERIAL**

**Supplementary Table S1 – Details relating to the sixteen centres and their referral patterns**

| Centre number | Approximate population (k) | Referrals screened | % wrist pain | Time for 10 referrals (days) |
| --- | --- | --- | --- | --- |
| 1 | 350 | 102 | 9.8% | 31 |
| 2 | 500 | 100 | 10% | 98 |
| 3 | 590 | 161 | 6.2% | 124 |
| 4 | 500 | 65 | 15.3% | 140 |
| 5 | 800 | 68 | 14.7% | 58 |
| 6 | 360 | 200 | 5% | 129 |
| 7 | 850 | 69 | 14.4% | 114 |
| 8 | 350 | 38 | 26.3% | 15 |
| 9 | 1000 | 106 | 9.4% | 90 |
| 10 | 550 | 103 | 9.7% | 115 |
| 11 | 300 | 361 | 2.8% | 330 |
| 12 | 650 | 61 | 16.4% | 19 |
| 13 | 350 | 291 | 34.4% | 171 |
| 14 | 350 | 68 | 14.7% | 46 |
| 15 | 600 | 85 | 11.8% | 100 |
| 16 | 500 | 171 | 5.8% | 121 |
| Overall (mean unless otherwise stated) | 538  8600 total | 125  1878 total | 12.9% | 106 |

**Supplementary Table S2 Detailed diagnostic breakdown of included patients**

| Centre number | 1 | 2 | 3 | 4 | 5 | 6 | 7 | 8 | 9 | 10 | 11 | 12 | 13 | 14 | 15 | 16 | Totals | % of all cases |
| --- | --- | --- | --- | --- | --- | --- | --- | --- | --- | --- | --- | --- | --- | --- | --- | --- | --- | --- |
| 1. OA wrist |  | 5 |  | 3 | 3 | 6 | 2 | 5 | 3 | 4 | 2 | 3 | 3 | 5 | 2 | 2 | 48 | 30% |
| - 1. SLAC/SNAC |  | 1 |  | 1 | 1 | 1 |  |  | 2 | 2 |  |  |  | 3 |  | 1 | 12 |  |
| - 1. pancarpal |  | 1 |  |  |  | 1 |  | 3 |  |  |  |  |  |  |  |  | 5 |  |
| - 1. radiocarpal |  | 2 |  |  | 1 | 2 | 2 | 1 | 1 | 1 | 1 | 1 |  |  | 1 | 1 | 14 |  |
| - 1. Other – STTJ/PT |  |  |  | 2 |  | 1 |  | 1 |  |  | 1 | 1 | 2 | 2 | 1 |  | 11 |  |
| e. DRUJ |  | 1 |  |  | 1 | 1 |  |  |  | 1 |  | 1 | 1 |  |  |  | 6 |  |
| 1. Ulnar sided | 3 | 3 | 5 |  | 1 | 3 |  |  | 1 | 5 | 1 | 1 | 1 | 3 | 3 |  | 30 | 18.8% |
| - 1. Abutment/ulnocarpal OA | 1 | 1 | 2 |  |  | 1 |  |  |  | 1 | 1 |  |  | 1 | 2 |  | 10 |  |
| - 1. TFCC tear |  | 1 | 3 |  | 1 | 2 |  |  | 1 | 3 |  | 1 | 1 | 1 | 1 |  | 15 |  |
| - 1. ECU tendinopathy | 2 | 1 |  |  |  |  |  |  |  | 1 |  |  |  | 1 |  |  | 5 |  |
| 1. Tendinopathy | 3 |  |  | 3 | 1 |  | 1 | 1 | 3 | 1 | 2 | 3 | 2 |  | 1 | 3 | 24 | 15% |
| - 1. 1^st^ | 3 |  |  | 3 | 1 |  |  | 1 | 3 | 1 | 2 | 3 |  |  | 1 | 3 | 21 |  |
| - 1. other |  |  |  |  |  |  | 1 |  |  |  |  |  | 2 |  |  |  | 3 |  |
| 1. Ganglion | 3 | 1 | 2 | 3 |  |  | 3 | 1 |  |  |  |  | 1 |  | 1 | 4 | 18 | 11.2% |
| 1. Other |  |  |  |  |  |  |  |  |  |  |  |  |  |  |  |  | 40 | 25% |
| - 1. Instability |  |  | 1 |  | 3 |  | 1 |  |  |  | 1 |  | 1 | 2 | 1 |  | 10 |  |
| - 1. AVN | 1 |  |  |  | 1 |  |  | 1 |  |  | 1 |  | 1 |  |  | 1 | 6 |  |
| - 1. Non-specific/unknown |  | 1 | 2 | 1 | 1 |  | 3 | 2 | 3 |  | 3 | 3 | 1 |  | 1 |  | 21 |  |
| - 1. Other |  |  |  |  |  | 1 |  |  | 1 |  |  |  |  |  | 1 |  | 3 |  |
| OA: osteoarthritis; SLAC: scapholunate advanced collapse; SNAC: scaphoid non-union advanced collapse; TFCC: triangular fibrocartilage complex; ECU: extensor carpi ulnaris; 1^st:^ De Quervain’s; AVN: avascular necrosis | | | | | | | | | | | | | | | | | | |
